# Supplementary material for: Cellular interpretation of the long-range gradient of Four-jointed activity in the Drosophila wing
Source: eLife. 2015 Feb 24;4:e05789. doi: 10.7554/eLife.05789 (PMC4338440; doi:10.7554/eLife.05789)
Supplement: Supplementary file 1. — Table contains numbers of wings, plateau data, and 95% confidence of plateau data for each FRAP experiment. DOI: http://dx.doi.org/10.7554/eLife.05789.019 [file elife05789s001.doc]

**Supplementary file 1**

Additional data for each FRAP experiment. Table contains numbers of wings for each experiment, plateau data and 95% confidence of plateau data.

| **Genotype** | **Number of Wings** | **Plateau** | **95% Confidence Interval of Plateau** |
| --- | --- | --- | --- |
| *ds-EGFP* puncta | 4 | 0.5307 | 0.5036 to 0.5579 |
| *ds-EGFP* non-puncta | 5 | 0.7704 | 0.7372 to 0.8037 |
| *ft-EGFP* puncta | 6 | 0.5618 | 0.5381 to 0.5855 |
| *ft-EGFP* non-puncta | 6 | 0.7347 | 0.7058 to 0.7637 |
| *ds-EGFP ft-^Grv^* clones | 2 | 0.8863 | 0.8385 to 0.9342 |
| *ft-EGFP ds^UA071^* | 2 | 0.8698 | 0.8217 to 0.9179 |
| *ds-EGFP fj^d1^* puncta | 4 | 0.5931 | 0.5573 to 0.6288 |
| *ds-EGFPfjd1* non-puncta | 4 | 0.8742 | 0.8340 to 0.9144 |
| *ft-EGFPfjd1* puncta | 3 | 0.7502 | 0.7225 to 0.7778 |
| *ft-EGFPfjd1* non-puncta | 3 | 0.8126 | 0.7800 to 0.8452 |
| *Act-ds-EGFP* | 5 | 0.6920 | 0.6703 to 0.7136 |
| *Act-ds-^S>Ax3^-EGFP* | 4 | 0.4660 | 0.4534 to 0.4786 |
| *Act-ds-^S>Dx3^-EGFP* | 5 | 0.6811 | 0.6575 to 0.7048 |
| *fj^-^ Act-ds-EGFP* | 5 | 0.6433 | 0.6207 to 0.6659 |
| *fj^-^Act-ds-EGFP ^S>Ax3^-EGFP* | 5 | 0.7047 | 0.6828 to 0.7266 |
| *fj^-^Act-ds-^S>Dx3^-EGFP* | 5 | 0.7004 | 0.6759 to 0.7249 |
| *Act-ft-EGFP* | 4 | 0.7081 | 0.6960 to 0.7202 |
| *Act-ft-^S/T>Ax5^-EGFP* | 7 | 0.7748 | 0.7632 to 0.7864 |
| *Act-ft-^S/T>Dx4^-EGFP* | 6 | 0.7360 | 0.7246 to 0.7474 |
| *fj^-^Act-ft-EGFP* | 4 | 0.7632 | 0.7480 to 0.7783 |
| *fj^-^Act-ft-^S/T>Ax5^-EGFP* | 2 | 0.8106 | 0.7811 to 0.8401 |
| *ds-EGFP* puncta (low Fj/proximal) | 4 | 0.5307 | 0.5036 to 0.5579 |
| *ds-EGFP* puncta (high Fj/distal) | 4 | 0.3534 | 0.2911 to 0.4156 |
| *fj^-^ds-EGFP* puncta (low Fj/proximal) | 4 | 0.5931 | 0.5573 to 0.6288 |
| *fj^-^ds-EGFP* puncta (high Fj/distal) | 4 | 0.4011 | 0.3614 to 0.4408 |
| *Act-ds-^S>Ax3^-EGFP* (low Fj/proximal) | 4 | 0.6899 | 0.6687 to 0.7112 |
| *Act-ds-^S>Ax3^-EGFP* (high Fj/distal) | 4 | 0.6362 | 0.6156 to 0.6567 |
| *fj^-^Act-ds-EGFP ^S>Ax3^-EGFP* (low Fj/proximal) | 4 | 0.6944 | 0.6802 to 0.7087 |
| *fj^-^Act-ds-EGFP ^S>Ax3^-EGFP* (high Fj/distal) | 3 | 0.8880 | 0.8668 to 0.9093 |
| *ds-EGFP* puncta distal pre-pupal wing | 4 | 0.5259 | 0.4852 to 0.5665 |
| *ds-EGFP* non-puncta distal pre-pupal wing | 4 | 0.8685 | 0.8073 to 0.9297 |
| *ft-EGFP ds^UA071^* Re-Bleach | 4 | 0.9461 | 0.8971 to 0.9951 |
